# Gapless genome assembly of East Asian finless porpoise

**DOI:** 10.1038/s41597-022-01868-4

**Published:** 2022-12-13

**Authors:** Denghua Yin, Chunhai Chen, Danqing Lin, Jialu Zhang, Congping Ying, Yan Liu, Wang Liu, Zhichen Cao, Chenxi Zhao, Chenhe Wang, Liping Liang, Pao Xu, Jianbo Jian, Kai Liu

**Affiliations:** 1 Key Laboratory of Freshwater Fisheries and Germplasm Resources Utilization, Ministry of Agriculture and Rural Affairs, Freshwater Fisheries Research Center, Chinese Academy of Fishery Sciences, Wuxi, 214081 China; 2 BGI Genomics, BGI-Shenzhen, Shenzhen, 518083 China; 3 Wuxi Fisheries College, Nanjing Agricultural University, Wuxi, 214081 China; 4 National Demonstration Center for Experimental Fisheries Science Education, Shanghai Ocean University, Shanghai, 201306 China

**Keywords:** Phylogenetics, Conservation biology

## Abstract

In recent years, conservation efforts have increased for rare and endangered aquatic wildlife, especially cetaceans. However, the East Asian finless porpoise (*Neophocaena asiaeorientalis sunameri*), which has a wide distribution in China, has received far less attention and protection. As an endangered small cetacean, the lack of a chromosomal-level reference for the East Asian finless porpoise limits our understanding of its population genetics and conservation biology. To address this issue, we combined PacBio HiFi long reads and Hi-C sequencing data to generate a gapless genome of the East Asian finless porpoise that is approximately 2.5 Gb in size over its 21 autosomes and two sex chromosomes (X and Y). A total of 22,814 protein-coding genes were predicted where ~97.31% were functionally annotated. This high-quality genome assembly of East Asian finless porpoise will not only provide new resources for the comparative genomics of cetaceans and conservation biology of threatened species, but also lay a foundation for more speciation, ecology, and evolutionary studies.

**Table:** 

Measurement(s)	*Neophocaena asiaeorientalis sunameri* • Gapless genome assembly • sequence annotation
Technology Type(s)	MGISEQ. 2000 • PacBio HiFi Sequencing • Hi-C
Sample Characteristic - Organism	*Neophocaena asiaeorientalis sunameri*
Sample Characteristic - Environment	seawater
Sample Characteristic - Location	Yellow Sea near Lianyungang City, Jiangsu Province, China

## Background & Summary

The finless porpoise (*Neophocaena* spp.) is a group of small-sized, toothed whales that are mainly distributed in southern and eastern Asia. Their distribution includes the coastal waters of the western Pacific Ocean, Indian Ocean, Sea of Japan, and they also appear in the Bohai Sea, Yellow Sea, East China Sea, South China Sea, and middle and lower reaches of the Yangtze River in Chinese waters^1,2^. Since Cuvier first named the species *Delphinus phocaenoides* in 1829, the taxonomy and nomenclature of the finless porpoise have been controversial^3,4^. For decades, the finless porpoise was considered to be a single species consisting of three subspecies^5–7^, until Wang and Jefferson *et al*. concluded that the genus *Neophocaena* can be divided into two separate species based on their morphological and genetic characteristics, including the Indo-Pacific finless porpoise (*N. phocaenoides*) and the narrow-ridged finless porpoise (*N. asiaeorientalis*). The narrow-ridged finless porpoise can also be divided into two subspecies that include the Yangtze finless porpoise (*N. a. asiaeorientalis*) and the East Asian finless porpoise (*N. a. sunameri*)^8,9^, and this classification has been generally accepted. In 2018, Zhou *et al*. performed *de novo* genome sequencing of the Yangtze finless porpoise and re-sequenced three geographic populations in Chinese waters to investigate the freshwater adaptation mechanisms of the Yangtze finless porpoise^10^. Their results found that the genetic differentiation between the Yangtze finless porpoise and East Asian finless porpoise reached interspecific level, which supports their classification as independent species^10^.

With conservation, *The IUCN Red List of Threatened Species* categorized the Yangtze finless porpoise as “critically endangered” in 2013^11^, and the narrow-ridged finless porpoise as “endangered” in 2017^12^. However, the East Asian finless porpoise was not listed separately. The East Asian finless porpoise was listed in the Second Class of the National Key Protected Wild Animals List in China announced on February 5, 2021. Similar to other small cetaceans throughout the world, the East Asian finless porpoise population faces many critical factors, such as marine environment pollution, fishing injury, loss of important habitat, and decline of fish resources under the dual influence of global climate change and human activities^13^. Ultimately, the prospect of East Asian finless porpoise increasing in population is not optimistic, and it is extremely urgent to explore more conservation efforts for this species.

**Fig. 1:**
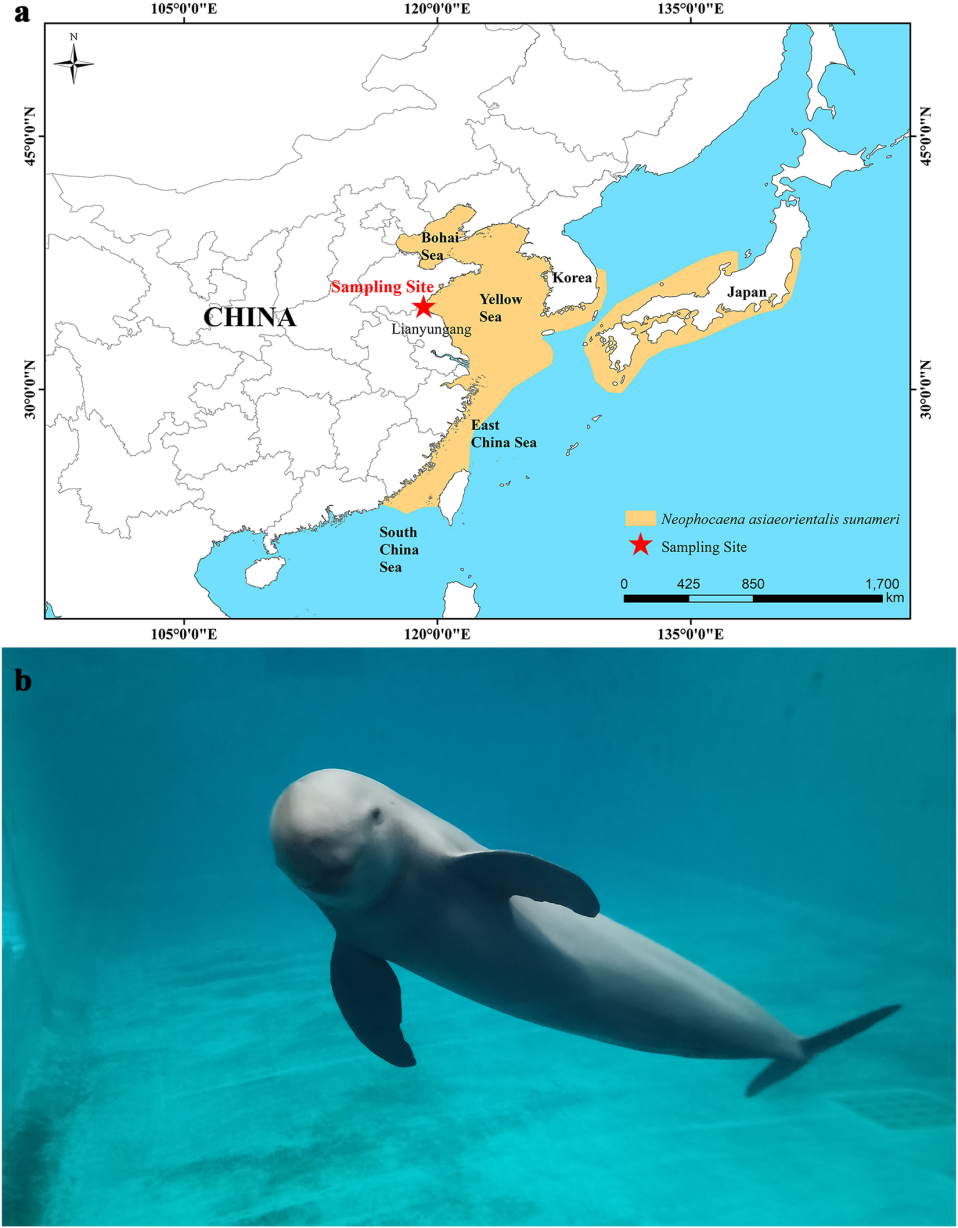
Location distribution and photograph of the East Asian finless porpoise, *N. a. sunameri*. (**a**) A natural distribution map of *N. a. sunameri* and the sampling site (red star) of the study. (**b**) *N. a. sunameri* photographed in Penglai Polar Ocean World, Penglai, Shandong, China.

With regards to field research, East Asian finless porpoises are mainly found in the temperate waters of the west coast of the Pacific Ocean. For example, the coastal waters from the Taiwan Strait through the East China Sea north to the Yellow Sea and the Bohai Sea in China, as well as the waters of Korea and Japan^7^ (Fig. 1). Their wide distribution area makes it incredibly difficult to conduct systematic survey assessments for the entire population. Therefore, their total population size has not been accurately reported, which is unfavorable for their conservation. Because of these issues, population numbers are only recorded in local areas. Yoshida, Amano and Shirakihara assessed the population size of East Asian finless porpoise in the Ariake Sound/Tachibana Bay, Chiba/Sendai Bay and the Inland Sea of Japan, and estimated the population size to be 7,572 in the Inland Sea of Japan^14–16^. Population evaluation of the East Asian finless porpoise in China is only found in the Bohai Sea^17^.

Compared to Yangtze finless porpoise, very few systematic studies have explored the molecular biology, ecology, acoustics and feeding behavior of the East Asian finless porpoise over the past 40 years. Ruan *et al*. sequenced and compared the renal transcriptomes between the Yangtze finless porpoise and the East Asian finless porpoise to investigate the mechanism of osmotic pressure regulation with adaptation to their different habitats^18^. Additionally, Li *et al*. used a single hydrophone to record and analyze the echolocation signals of East Asian finless porpoises in Liaodong Bay and conducted a comparative study to Yangtze finless porpoises^19^. Further, Dong *et al*. concluded that the migration pattern of the East Asian finless porpoise population is mainly related to the migratory distribution of its preferred fish^20^, and finless porpoises have a broad diet that largely consists of fish, shrimp, and cephalopods^21,22^. Although these studies help understand finless porpoise migration behaviors and adaptation, more research needs to focus on improving conservation efforts for the East Asian finless porpoise.

In China, conservation research on the East Asian finless porpoise is less enthusiastic than that on the Yangtze finless porpoise. There is a serious lack of basic research on the East Asian finless porpoise, especially on its current population size, distribution characteristics, migration patterns, and key habitats. To date, the population size and distribution pattern of the East Asian finless porpoise in China are still not systematically known, and little is known about its key habitats. Consequently, its conservation biology research should receive more attention because it is an endangered marine mammal that is also listed as a second-class key protected wild animal in China.

The goal of this study was to assemble a gapless genome for the East Asian finless porpoise to aid in the conservation of this species. Here, we report a gapless cetacean genome that was generated through combining PacBio HiFi long reads and Hi-C sequencing data. We sequenced and analyzed the genome of the East Asian finless porpoise at the chromosomal level to gain a deeper understanding of its genetic background and evolutionary characteristics. The assembled genome size is approximately 2.50 Gb with a contig N50 of 84.69 Mb and scaffold N50 of 122.40 Mb. A total of 52 contigs were anchored onto 23 chromosomes (21 autosomes, X and Y chromosomes), and one mitochondrion chromosome. This genome contained 7 gapless assemblies of chromosome 4 (147 Mb), chromosome 9 (108 Mb), chromosome 11 (102 Mb), chromosome 16 (86 Mb), chromosome 18 (80 Mb), chromosome 21 (36 Mb), and chromosome X (125 Mb). Consequently, only 28 gaps were retained for next step filling in our assembly results. As the telomere-to-telomere assembly of human genome published this year^23^, ultra-long (>100-kbp) nanopore reads can be enable to span complex repeats and complete assemblies of the centromeres and telomeres. Gene annotation yielded 22,814 protein-coding genes and 97.31% of the predicted genes were annotated in publicly available biological databases, including NR, GO, KOG, KEGG, TrEMBL, Interpro and Swissprot. This high-quality assembled genome will provide rich research resources for conservation biology and phylogenetic studies on the East Asian finless porpoise, as well as research on genetic differentiation and adaptive evolution of other small toothed whales, like the Yangtze finless porpoise.

## Methods

### Sample collection

A muscle sample was collected from a male specimen of East Asian finless porpoise that died in the Yellow Sea near Lianyungang City, Jiangsu Province, China, in 2019 (Fig. 1). No ethical issues were considered in this study. The muscle sample was washed 3 times with Phosphate buffer saline (PBS), quickly frozen in liquid nitrogen, and stored at −80 °C until DNA extraction.

### WGS library construction and genome size estimation

DNA was extracted from muscle specimen of the East Asian finless porpoise using MZ 1.3 (hypervariable minisatellite probe), as well as locus-specific minisatellite probes (g3, MS1 and MS43). For the short insert WGS library, DNA was sheared into fragments between 50 to 800 bp using a Covaris E220 ultrasonicator (Covaris, Brighton, UK) according to the manufacturer’s instructions. Fragments between 300 to 400 bp were selected to generate a single-stranded circular DNA library. The DNA library was sequenced on a MGISEQ-2000 platform. A total of 232.16 Gb of raw short reads were generated and 182.87 Gb of clean data were retained after adaptor removing and low-quality reads filtering by SOAPnuke (v2.0)^24^ with parameters “-n 0.01 -l 20 -q 0.1 -i -Q 2 -G 2 -M 2 -A 0.5” (Supplementary Table S1).

**Fig. 2:**
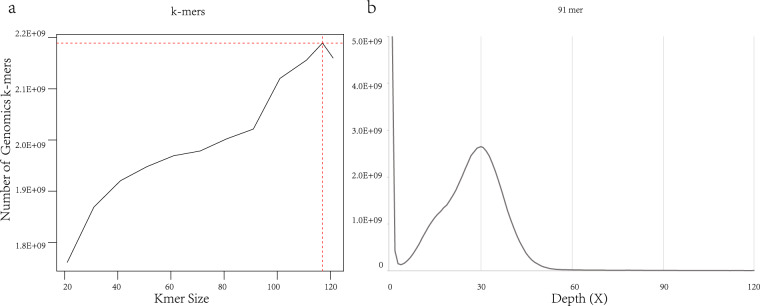
K-mers analysis to estimate the genome size of the East Asian finless porpoise. (**a**) 182 Gb of high-quality data was used to generate 16 different k-mers depth distribution curve frequencies by KmerGenie. The k-mers value was automatically set by the software from 21 to 121. The x-axis indicates k-mer size, while the y-axis is the number of genomics k-mers at that k-mer size. (**b**) 91-mer depth frequency distribution. The x-axis is depth (X), while the y-axis is the proportion that represents the frequency at that depth divided by the total frequency of all depths. The genome size was estimated using the following formula: Genome size = K-mer num/Peak depth. The peak depth is approximately 28 and the estimated genome size is 2,475,638,739 bp.

We used KmerGenie (v1.7051)^25^ to estimate the genome size with varied k-mer sizes from 21 to 121 (Fig. 2a). According to the smooth curves of estimated genome size, we obtained the predicted optimal k-mer size of 91 and the predicted genome size of 2,475,638,739 bp (Fig. 2b). The predicted genome size of the East Asian finless porpoise is consistent with that of the Yangtze finless porpoise (2.49 Gb) found in a previous study^10^.

### PacBio library preparation, sequencing, and de novo assembly using HiFi reads

DNA was extracted from the same muscle specimen using a QIAGEN Blood & Cell Culture DNA Midi Kit following the manufacturer’s instruction (QIAGEN, Germany). After DNA extraction, two sequencing libraries were prepared according to the “Using SMRTbell Express Template Prep Kit 2.0 With Low DNA Input” protocol from PacBio with an insert size of approximately 20 kb (Pacific Biosciences, USA). The libraries were then sequenced on a PacBio Sequel II SMRT cells in circular consensus sequence (CCS) mode. A total of 5 SMRT Cells were sequenced. 2,397 Gb subreads were processed using the CCS algorithm of SMRTLink (v8.0.0)^26^ with parameters “–minPasses 3 –minPredictedAccuracy 0.99 –minLength 500”, yielding 154 Gb of PacBio’s long high-fidelity (HiFi) reads (Supplementary Table S1). With the HiFi reads, the primary contigs were assembled by Hifiasm (v0.15.1)^27^ with default parameters. After, the Purge-Haplotigs^28^ program was used to remove redundant sequences with parameters “-j 80 -s 80 -a 30”, which yielded a contig assembly with a size of approximately 2.50 Gb and contig N50 of 84.69 Mb (Table 1).

**Table 1:** Statistics of assembly.

Statistical level	Super-scaffold length (bp)	Number	Contig length (bp)	Number
Total number		24		52
Total length (bp)	2,497,855,621		2,497,841,621	
Gap number (bp)	14,000	28	0	0
N50	122,398,165	8	84,691,504	13
N90	80,211,367	19	29,542,604	32
Maximum length (bp)	210,684,203		147,659,217	
Minimum length (bp)	30,528		27,689	
GC content (%)		41.70%		41.70%
BUSCO Evaluation (mammalian)	C:95.4%[S:93.7%,D:1.7%],F:1.1%,M:3.5%,n:9226		C:95.2%[S:93.4%,D:1.8%],F:1.3%,M:3.5%,n:9226	

### Hi-C library preparation, sequencing, and chromosome anchoring

The same muscle specimen was fixed with 1% formaldehyde for 10–30 min at room temperature to coagulate proteins that are involved in chromatin interaction in the genome. The restriction enzyme Mbo I (NEB, Ipswich, USA) was then added to digest the DNA, and fragments with flat or sticky ends were obtained. The ends were flattened and repaired, and then labeled with biotin. The inter-match fragments were ligated with T4 DNA ligase (Thermo Scientific, USA) to form a loop. Proteins that connected the DNA fragments were then digested to obtain the crosslinked fragments, and the clip was interrupted again using ultrasound. A Hi-C library was made by capturing the biotin with magnetic beads and sequenced on a MGISEQ-2000 instrument. A total of 219.2 Gb of clean data were obtained from 263.87 Gb of sequencing data using software SOAPnuke (v2.0)^24^ with parameters “-n 0.01 -l 20 -q 0.1 -i -Q 2 -G 2 -M 2 -A 0.5” (Supplementary Table S1).

**Fig. 3:**
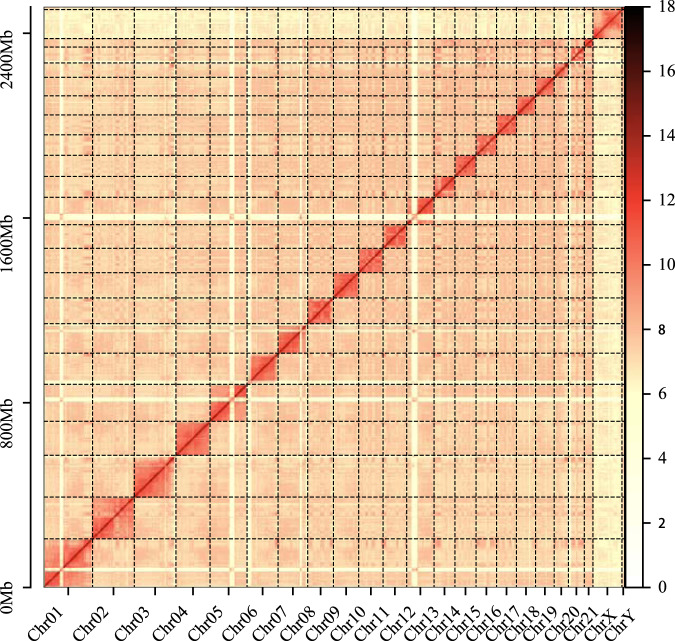
Genome-wide Hi-C heat maps of the East Asian finless porpoise genome.

To anchor contigs onto chromosomes, the Hi-C clean data were mapped to the assembled contigs using BWA (v0.7.12)^29^, and then erroneous mappings (MAPQ = 0) and duplicates were filtered by the juicer pipeline (v1.5)^30^ to obtain the interaction matrix. Following, approximately 625.70 Mb reads (~77.77%) were used to anchor the contigs into chromosomes with 3D-DNA pipeline (v180,922)^31^. And 3D-DNA pipeline^31^ was used to remove select short contigs using default parameters. The Hi-C contact maps were then reviewed with JUICEBOX Assembly Tools (v2.15.07)^30^. These processes generated a final genome assembly, where the genome size was approximately 2.50 Gb and contig N50 was 84.69 Mb. Remarkably, 52 contigs were linked onto the 21 autosomes, two sex chromosomes, and one mitochondria sequence (Fig. 3, Tables 1 and 2).

**Table 2:** Chromosomes Length of East Asian finless porpoise.

ID	Length (bp)	N%	GC%	Contig Number	Gap Number
Chr01	210,684,203	0	42.41	6	5
Chr02	180,789,114	0	41.57	3	2
Chr03	180,234,385	0	41.12	3	2
Chr04	147,659,217	0	39.5	1	0
Chr05	160,011,187	0	39.86	2	1
Chr06	122,398,165	0	42.1	2	1
Chr07	127,996,830	0	40.55	4	3
Chr08	111,548,431	0	42.51	3	2
Chr09	108,601,407	0	40.45	1	0
Chr10	105,006,622	0	43.17	2	1
Chr11	102,703,641	0	41.69	1	0
Chr12	117,621,280	0	40.99	3	2
Chr13	90,542,439	0	42.83	2	1
Chr14	91,315,151	0	41.74	3	2
Chr15	88,758,320	0	45.88	2	1
Chr16	86,011,275	0	42.87	1	0
Chr17	82,389,581	0	40.76	2	1
Chr18	80,211,367	0	39.43	1	0
Chr19	61,752,428	0	45.74	3	2
Chr20	68,815,040	0	46.24	2	1
Chr21	36,121,570	0	40.92	1	0
X	125,630,717	0	40.24	1	0
Y	11,022,723	0	43.51	2	1
MT	30,528	0	40.76	1	0

### Identification of Y chromosome sequences

Generally, sequence assembly on the Y chromosome is a challenge due to its complex repetitive nature. Here, we assembled all the PacBio HiFi reads into contigs by Hifiasm^27^ software, and then filtered the redundant sequences using Purge-Haplotigs^28^ software. Finally, we anchored the non- redundant contigs into scaffold with Hi-C data. In order to identify the Y sequence, we mapped the scaffold sequences onto the Y chromosome of *Tursiops truncates*^32^. Additionally, we mapped the contig sequences of the East Asian finless porpoise genome into Y chromosome of *Tursiops truncates* genome using Ragtag (v2.1.0)^33^ with default parameters. Based on the above two methods, we obtained a candidate Y sequence with a high degree of similarity. We selected the Y sequence assembled from the first method. The newly assembled Y chromosome sequence is 11.02 Mb in length and contains 82 intact protein-coding gene models. Of these 82 genes, seven genes were identified as TSPY genes. In humans, these four genes (SRY, TSPY1, TSPY3 and ZFY) are linked to the Y chromosome^34–39^. Accordingly, this assembled Y chromosome sequence is highly reliable.

### Repeat annotation

Two strategies including *de novo* and homolog methods were used to annotate repeat elements. *De novo* repeats were identified by RepeatModeler (v1.0.4)^40^ and long terminal repeats were annotated by LTR-FINDER (v1.0.7)^41^. DNA and protein transposable elements (TEs) were detected by RepeatMasker (v4.0.7)^42^ and RepeatProteinMasker (v4.0.7), respectively, based on Repbase database^43^. Tandem repeats were performed by Tandem Repeat Finder (v4.10.0)^44^. We obtained approximately 1.05 Gb (~42.23%) of repetitive sequences (Supplementary Table S2), which were similar to the Yangtze finless porpoise^10^, and 38.63% belonged to LINE subfamily (Supplementary Table S3).

### Protein-coding genes prediction and functional annotation

**Fig. 4:**
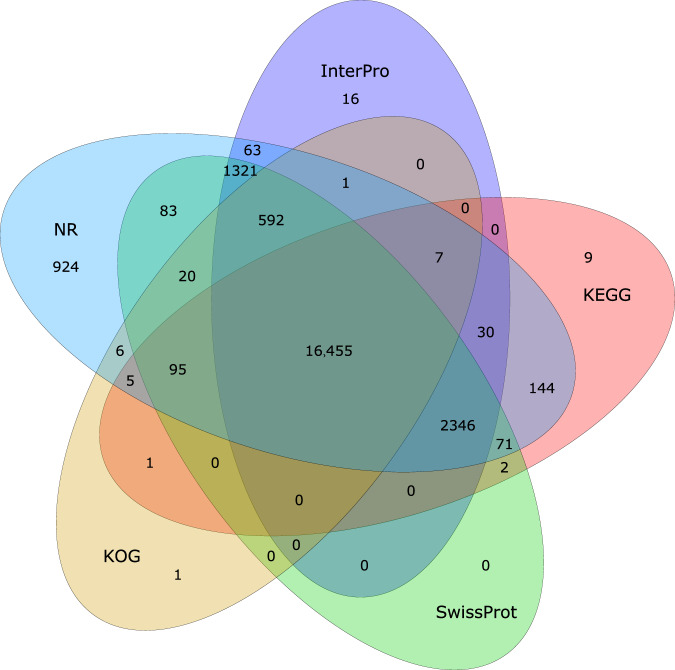
Gene function annotation results in a statistics Venn diagram using five public databases: NR, InterPro, KEGG, SwissProt and KOG.

To predict genes, we generated 32 RNA-seq samples from blood tissue of the Yangtze finless porpoise specimen (Supplementary Table S4)^45,46^. These reads were then aligned to East Asian finless porpoise genome using Hisat2 (v2.1.0)^47^ with the following parameters: –sensitive –no-discordant –no-mixed -I 1 -X 1000 –max-intronlen 1000000. The aligned reads were assembled using Stringtie (v1.3.5)^48^ with the following parameters: -f 0.3 -j 3 -c 5 -g 100 -s 10000. Subsequently, TransDecoder (v5.5.0) (https://github.com/TransDecoder/TransDecoder) was used to identify the coding sequence with default parameters. Gene models for the East Asian finless porpoise were also predicted by Augustus (v3.2.1)^49^ for *de novo* annotation. Homologous proteins of eight reference species were downloaded from common databases. Data for the Bowhead whale^50^ was downloaded from the Bowhead Whale Genome Resource (http://www.bowhead-whale.org). Common minke whale^51^, Beluga whale^52^, Yangtze River dolphin^53^, Yangtze finless porpoise^10^, Killer whale^54^, Bottlenose dolphin^54^ and Sperm whale were downloaded from the National Center for Biotechnology Information (NCBI). GeMoMa (v1.8)^55^ was used to search coding structures based on transcriptome data and homologous proteins. A total of 22,814 coding genes (36,167 transcripts) were predicted (Supplementary Table S5). All protein coding genes were supported by at least one prediction method (Supplementary Table S6). The final gene set was functionally annotated by mapping against KEGG^56^, Swiss-Prot^57^, TrEMBL^57^, KOG^58^, InterPro^59^ and NR (NCBI Non-redundant protein) databases using BLAST (v2.2.26)^60^ with an E-value threshold of 1E-5. The protein domains and motifs were annotated using InterProScan^61^. GO Ontology (GO)^62^ was obtained from the InterProScan^61^ results in this study, and 97.31% of the 22,814 proteins were annotated by at least one database (Supplementary Table S7). Of these functional proteins, 16,455 (~72%) were supported by all five databases (Fig. 4).

## Data Records

The DNA sequence reads of East Asian finless porpoise (Experiment of DNA sequencing data from genome survey library: SRR21047154^63^; Experiments of DNA sequencing data from Hi-C library: SRR20760935^64^-SRR20760936^65^; Experiments of DNA sequencing data from PacBio HiFi library: SRR20997931-SRR20997935^66–70^) have been deposited in the Sequence Read Archive (SRA) under project number SRP389529^71^. The Whole Genome Shotgun project has been deposited at DDBJ/ENA/GenBank under the accession JANJGR000000000^72^. Files of the assembled genome, gene structure annotation, repeat predictions and gene functional annotation of East Asian finless porpoise were deposited at Figshare database under DOI code^73^.

## Technical Validation

### Evaluation of the genome assembly

By comparing the assembled metrics of the East Asian finless porpoise to the other cetacean species, our assembly substantially improved because of increased contig and scaffold lengths, which indicates that our assembly is highly contiguous. Our gapless genome assembly increased the contiguity metrics 941-fold (by contig N50) or 921-fold (by the number of contigs) compared to a previously reported Yangtze finless porpoise assembly^10^. Among the public cetacean genomes, our assembly had the longest contig N50 length and smallest gap number, which suggests that our East Asian finless porpoise genome is high quality (Table 3).

**Table 3:** Comparison of the East Asian finless porpoise genome with previously published cetacean genomes.

Species	Genome Size/Gb	Contig N50/Mb	Contig Number	Super-scaffold N50/Mb	BUSCO Evaluation	Data Source	Sequencing Technology
East Asian finless porpoise	2.50	84.69	52	122.40	C:95.4%	This study	Pacbio Sequel II HiFi; HiC
Bottlenose dolphin	2.37	9.73	1,003	108.43	C:94.9%	GCF_011762595	PacBio Sequel I CLR; Illumina NovaSeq; Arima Genomics Hi-C; Bionano Genomics DLS
Beluga whale	2.32	0.20	23,189	31.18	C:95.0%	GCF_002288925	Illumina HiSeqX
Indo-Pacific humpback dolphin	2.45	0.11	113,866	27.70	C:91.9%	GCA_007760645	Illumina HiSeq
Yangtze finless porpoise	2.27	0.09	47,942	6.34	C:94.0%	GCF_000442215	Illumina Hiseq. 2000
Killer whale	2.23	0.07	59,440	12.74	C:95.0%	GCF_000331955	Illumina HiSeq
Bowhead whale	2.07	0.04	96,651	0.88	C:90.0%	bowhead-whale.org	Illumina HiSeq. 2000
Sperm whale	2.37	0.04	94,454	122.18	C:92.3%	GCF_002837175	BGISEQ-500
Yangtze River dolphin	2.37	0.03	118,297	2.44	C:94.6%	GCF_003031525	Illumina HiSeq. 2000
Common minke whale	2.26	0.02	147,744	13.03	C:93.8%	GCF_000493695	Illumina HiSeq. 2000

To assess the completeness of our East Asian finless porpoise genome, we performed an analysis using Benchmarking Universal Single-Copy Orthologs (BUSCO, v5.1.0)^74^ with the mammalia_odb10 database. The results showed that 95.4% of the expected mammalian genes (including 93.7% single and 1.7% duplicated ones) had complete gene coverage, and 1.1% were identified as fragmented, respectively. However, 3.5% were considered missing in our East Asian finless porpoise genome. Still, the complete evaluation of the East Asian finless porpoise genome is more superior than other current public cetacean genomes (Table 3).

To evaluate the telomere sequences assembled in the East Asian finless porpoise genome, we used the Telomere Identification toolkit (Tidk, v0.2.0) (https://github.com/tolkit/telomeric-identifier) to search telomere sequences (TTAGGG) along with the genome sequence. From the results, 23 chromosomes detected at least one side of telomere sequences, such as Chr5 and Chr11. Individual sequences were identified with partial telomere sequences, which should be further investigated and optimized.

**Fig. 5:**
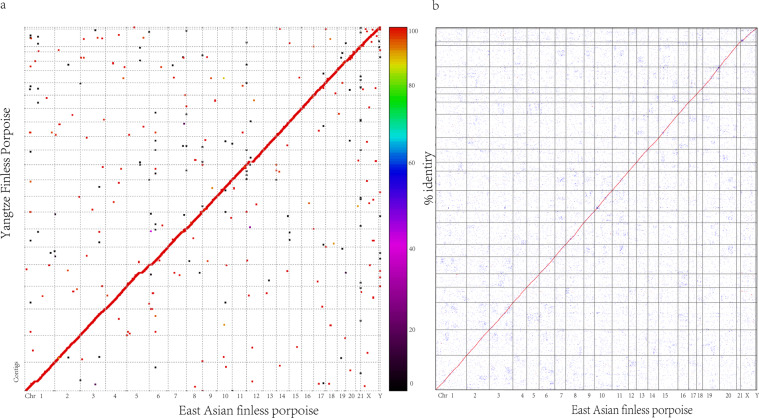
Comparison of sequence synteny between the East Asian finless porpoise and the Yangtze finless porpoise. (**a**) MUMmer was used to identify similar regions between the East Asian finless porpoise and Yangtze finless porpoise genome sequences. (**b**) WGDI was used to detect syntenic blocks between the East Asian finless porpoise and Yangtze finless porpoise gene pairs. The x-axis is the chromosome scale of East Asian finless porpoise genome, and the y-axis is the contig scale of Yangtze finless porpoise genome.

To compare the genome consistency between the East Asian finless porpoise and the Yangtze finless porpoise, we used MuMmer (v4.0.0)^75^ to identify similar regions with parameters “–mum -c 500 -l 40” at the genome level. Additionally, we also used BLAST^60^ and WGDI (https://github.com/SunPengChuan/wgdi) software to search the synteny blocks with at least ten gene pairs at the gene level. These two analyses revealed that the two genomes are highly consistent (Fig. 5).

### Evaluation of the gene annotation

We performed BUSCO^74^ analysis with the mammalia_odb10 database to assess the completeness of the coding sequences for the East Asian finless porpoise. The results showed that 97.9% of the expected mammalian genes (including 96.7% single and 1.2% duplicated ones) had complete gene coverage, and only 0.5% were identified as fragmented, respectively. However, 1.6% were considered missing in our East Asian finless porpoise genome. Compared to other complete evaluations of protein-coding genes, our East Asian finless porpoise has a high degree of integrity (Table 4).

**Table 4:** Comparison of the East Asian finless porpoise genes with other representative cetacean gene sets.

Species	Gene Number	Average mRNA length (bp)	Average CDS length (bp)	Average exon per gene	Average exon length (bp)	Average intron length (bp)	BUSCO
East Asian finless porpoise	22,814	64,616	2,035	10	211	5,053	C:97.9%[S:96.7%,D:1.2%],F:0.5%,M:1.6%
Common minke whale	18,400	50,782	1,697	10	165	4,702	C:93.9%[S:92.7%,D:1.2%],F:2.1%,M:4.0%
Bowhead whale	22,733	18,102	1,249	7	174	2,505	C:66.2%[S:65.3%,D:0.9%],F:12.9%,M:20.9%
Beluga whale	17,701	48,646	1,761	10	171	5,041	C:96.5%[S:95.7%,D:0.8%],F:0.7%,M:2.8%
Yangtze River dolphin	18,877	45,086	1,697	10	180	4,614	C:93.6%[S:93.1%,D:0.5%],F:0.9%,M:5.5%
Yangtze finless porpoise	18,479	36,930	1,687	10	166	3,842	C:94.6%[S:94.0%,D:0.6%],F:1.3%,M:4.1%
Killer whale	18,129	51,256	1,749	10	170	4,950	C:96.1%[S:94.6%,D:1.5%],F:0.8%,M:3.1%
Sperm whale	18,626	37,852	1,603	10	166	3,795	C:92.9%[S:92.1%,D:0.8%],F:2.8%,M:4.3%
Bottlenose dolphin	18,418	45,437	1,720	10	174	4,914	C:96.0%[S:94.3%,D:1.7%],F:0.9%,M:3.1%

## Supplementary information

Supplementary Tables

## Data Availability

No specific code was developed for this work. The data analyses were performed according to the manuals and protocols provided by the developers of the corresponding bioinformatics tools that are described in the Methods section together with the versions used.
