# Supplementary material for: Gapless genome assembly of East Asian finless porpoise
Source: Sci Data. 2022 Dec 13;9:765. doi: 10.1038/s41597-022-01868-4 (PMC9747978; doi:10.1038/s41597-022-01868-4)
Supplement: Supplementary file 1 — Supplementary Tables [file 41597_2022_1868_MOESM1_ESM.docx]

Supplementary Table legends

Supplementary Table 1 (Page 2) Summary of sequencing data for WGS library, PacBio HIFI library, and Hi-C library.

Supplementary Table 2 (Page 3) Summary of transposon element families in East Asian finless porpoise genome based on various methods.

Supplementary Table 3 (Page 4) Statistics of classified repeat in the East Asian finless porpoise genome.

Supplementary Table 4 (Page 5) Sample information of 32 blood samples of *Neophocaena asiaeorientalis asiaeorientalis* for gene structure annotation and expression analysis.

Supplementary Table 5 (Page 8) Summary of predicted protein-coding genes in the East Asian finless porpoise genome compared with other representative cetacean genomes.

Supplementary Table 6 (Page 9) The evidence supporting gene models of the East Asian finless porpoise genome.

Supplementary Table 7 (Page 10) Statistics of East Asian finless porpoise genes with functional classification by various methods.

**Table S1. Summary of sequencing data for** **WGS library, PacBio HIFI library, and Hi-C library.**

| **Pair-end Libraries** | **Insert Size** | **Average Length (bp)** | **Total Data (Gb)** | **Sequence Depth (X)** |
| --- | --- | --- | --- | --- |
| WGS | 300-400 bp | 150 | 182.87 | 73 |
| PacBio HIFI | 20 kb | 14,681 | 154.83 | 62 |
| Hi-C | 300-400 bp | 150 | 212.90 | 85 |

**Table S2. Summary of transposon element families in East Asian finless porpoise genome based on various methods.**

| Type | Repeat Length (bp) | % of genome |
| --- | --- | --- |
| Trf | 142,434,381 | 5.70 |
| Repeatmasker | 653,927,410 | 26.18 |
| Proteinmask | 285,287,012 | 11.42 |
| De novo | 965,675,596 | 38.66 |
| Total | 1,054,804,993 | 42.23 |

**Table S3. Statistics of classified repeat in the East Asian finless porpoise genome.**

| Type | | Length (bp) | % of genome |
| --- | --- | --- | --- |
| Retro | LTR/Copia | 70,844 | 0.00 |
| Retro | LTR/Gypsy | 380,114 | 0.02 |
| Retro | LTR/Other | 100,477,875 | 4.02 |
| Retro | SINE | 8,884,700 | 0.36 |
| Retro | LINE | 965,026,445 | 38.63 |
| Retro | Other | 0 | 0.00 |
| DNA | EnSpm | 594,826 | 0.02 |
|  | Harbinger | 533,944 | 0.02 |
|  | hAT | 14,255,435 | 0.57 |
|  | Helitron | 223,239 | 0.01 |
|  | Mariner | 3,004,161 | 0.12 |
|  | MuDR | 614,127 | 0.02 |
|  | P | 27,932 | 0.00 |
|  | Other | 30,779,322 | 1.23 |
| Other | - | 32,018,437 | 1.28 |
| Unknown | - | 935,960 | 0.04 |
| Total | - | 1,040,750,784 | 41.67 |

**Table S4. Sample information of 32 blood samples of *Neophocaena asiaeorientalis asiaeorientalis* for gene structure annotation and expression analysis.**

| **Sort** | **Sample** | **Species** | **Sex** | **Estimated Age** | **Accession Number** | **Assay_type** | **Tissue_type** | **Data_**  **type** | **Ageing** | **Sex** | **Habitat** | **Sample collection and collected time** |
| --- | --- | --- | --- | --- | --- | --- | --- | --- | --- | --- | --- | --- |
| 1 | CJ-1 | *N. a. asiaeorientalis* | Male | ＞13 year | PRJNA699632 | RNA-seq | blood | Ageing | Old |  |  | Sampling from main vein of the tail / 2017.11 |
| 2 | CJ-2 | *N. a. asiaeorientalis* | Male | ＞13 year | PRJNA699632 | RNA-seq | blood | Ageing | Old |  |  | Sampling from main vein of the tail / 2017.11 |
| 3 | CJ-3 | *N. a. asiaeorientalis* | Male | 13 year | PRJNA699632 | RNA-seq | blood | Ageing | Old |  |  | Sampling from main vein of the tail / 2017.11 |
| 4 | CJ-4 | *N. a. asiaeorientalis* | Male | 10 year | PRJNA699632 | RNA-seq | blood | Ageing | Adult |  | Natural water | Sampling from main vein of the tail / 2017.11 |
| 5 | CJ-5 | *N. a. asiaeorientalis* | Male | 10 year | PRJNA699632 | RNA-seq | blood | Ageing | Adult |  | Natural water | Sampling from main vein of the tail / 2017.11 |
| 6 | CJ-6 | *N. a. asiaeorientalis* | Male | 10 year | PRJNA699632 | RNA-seq | blood | Ageing | Adult |  | Natural water | Sampling from main vein of the tail / 2017.11 |
| 7 | CJ-7 | *N. a. asiaeorientalis* | Male | 6 year | PRJNA699632 | RNA-seq | blood | Ageing | Young | Male | Natural water | Sampling from main vein of the tail / 2017.11 |
| 8 | CJ-8 | *N. a. asiaeorientalis* | Male | 6 year | PRJNA699632 | RNA-seq | blood | Ageing | Young | Male | Natural water | Sampling from main vein of the tail / 2017.11 |
| 9 | CJ-9 | *N. a. asiaeorientalis* | Male | 6 year | PRJNA699632 | RNA-seq | blood | Ageing | Young | Male | Natural water | Sampling from main vein of the tail / 2017.11 |
| 10 | CJ-10 | *N. a. asiaeorientalis* | Female | 5 year | — | RNA-seq | blood | sex |  | Female |  | Sampling from main vein of the tail / 2017.11 |
| 11 | CJ-11 | *N. a. asiaeorientalis* | Female | 6 year | — | RNA-seq | blood | sex |  | Female |  | Sampling from main vein of the tail / 2017.11 |
| 12 | CJ-12 | *N. a. asiaeorientalis* | Female | 7 year | — | RNA-seq | blood | sex |  | Female |  | Sampling from main vein of the tail / 2017.11 |
| 13 | CJ-13 | *N. a. asiaeorientalis* | Male | 6 year | — | RNA-seq | blood | habitat |  |  | Natural water | Sampling from main vein of the tail / 2017.11 |
| 14 | CJ-14 | *N. a. asiaeorientalis* | Female | 10 year | — | RNA-seq | blood | habitat |  |  | Natural water | Sampling from main vein of the tail / 2017.11 |
| 15 | CJ-15 | *N. a. asiaeorientalis* | Male | 6 year | — | RNA-seq | blood | habitat |  |  | Natural water | Sampling from main vein of the tail / 2017.11 |
| 16 | XJ-1 | *N. a. asiaeorientalis* | Male | 10 year | — | RNA-seq | blood | habitat |  |  | Ex Situ Protected Water | Sampling from main vein of the tail / 2018.3 |
| 17 | XJ-2 | *N. a. asiaeorientalis* | Male | 10 year | — | RNA-seq | blood | habitat |  |  | Ex Situ Protected Water | Sampling from main vein of the tail / 2018.3 |
| 18 | XJ-3 | *N. a. asiaeorientalis* | Male | 9 year | — | RNA-seq | blood | habitat |  |  | Ex Situ Protected Water | Sampling from main vein of the tail / 2018.3 |
| 19 | XJ-4 | *N. a. asiaeorientalis* | Male | 5 year | PRJNA789349 | RNA-seq | blood | habitat |  |  | Ex Situ Protected Water | Sampling from main vein of the tail / 2018.3 |
| 20 | XJ-5 | *N. a. asiaeorientalis* | Male | 5 year | PRJNA789349 | RNA-seq | blood | habitat |  |  | Ex Situ Protected Water | Sampling from main vein of the tail / 2018.3 |
| 21 | XJ-6 | *N. a. asiaeorientalis* | Male | 5 year | PRJNA789349 | RNA-seq | blood | habitat |  |  | Ex Situ Protected Water | Sampling from main vein of the tail / 2018.3 |
| 22 | XJ-7 | *N. a. asiaeorientalis* | Male | 6 year | — | RNA-seq | blood | habitat |  |  | Ex Situ Protected Water | Sampling from main vein of the tail / 2018.5 |
| 23 | XJ-8 | *N. a. asiaeorientalis* | Female | 10 year | — | RNA-seq | blood | habitat |  |  | Ex Situ Protected Water | Sampling from main vein of the tail / 2018.5 |
| 24 | XJ-9 | *N. a. asiaeorientalis* | Male | 6 year | — | RNA-seq | blood | habitat |  |  | Ex Situ Protected Water | Sampling from main vein of the tail / 2018.5 |
| 25 | XJ-10 | *N. a. asiaeorientalis* | Female | 1 year | — | RNA-seq | blood | habitat |  |  | Ex Situ Protected Water | Sampling from main vein of the tail / 2018.5 |
| 26 | CL-1 | *N. a. asiaeorientalis* | Male | 7 year | — | RNA-seq | blood | habitat |  |  | Aquarium | Sampling from main vein of the tail / 2018.11 |
| 27 | CL-2 | *N. a. asiaeorientalis* | Female | 11 year | — | RNA-seq | blood | habitat |  |  | Aquarium | Sampling from main vein of the tail / 2018.11 |
| 28 | CL-3 | *N. a. asiaeorientalis* | Male | 7 year | — | RNA-seq | blood | habitat |  |  | Aquarium | Sampling from main vein of the tail / 2018.11 |
| 29 | CL-4 | *N. a. asiaeorientalis* | Male | 9 year | — | RNA-seq | blood | habitat |  |  | Aquarium | Sampling from main vein of the tail / 2020.10 |
| 30 | CL-5 | *N. a. asiaeorientalis* | Female | 13 year | — | RNA-seq | blood | habitat |  |  | Aquarium | Sampling from main vein of the tail / 2020.10 |
| 31 | CL-6 | *N. a. asiaeorientalis* | Male | 9 year | — | RNA-seq | blood | habitat |  |  | Aquarium | Sampling from main vein of the tail / 2020.10 |
| 32 | CL-7 | *N. a. asiaeorientalis* | Female | 3 year | — | RNA-seq | blood | habitat |  |  | Aquarium | Sampling from main vein of the tail / 2020.10 |

**Table S5. Summary of predicted protein-coding genes in the East Asian finless porpoise genome compared with other representative cetacean genomes.**

| Gene set | | Gene Number | mRNA Number | Average mRNA length (bp) | Average CDS length (bp) | Average exon per gene | Average exon length (bp) | Average intron length (bp) |
| --- | --- | --- | --- | --- | --- | --- | --- | --- |
| De novo | Augustus | 21,155 | 21,155 | 50,901 | 1,421 | 9 | 164 | 6,456 |
| Homolog | *B. acutorostrata* | 19,124 | 20,127 | 66,347 | 1,576 | 9 | 168 | 5,571 |
|  | *B. mysticetus* | 14,396 | 14,929 | 51,976 | 1,061 | 6 | 183 | 6,513 |
|  | *D. leucas* | 19,897 | 20,794 | 64,784 | 1,624 | 9 | 173 | 5,541 |
|  | *L. vexillifer* | 19,076 | 19,909 | 64,507 | 1,593 | 9 | 170 | 5,427 |
|  | *N. a. asiaeorientalis* | 19,616 | 20,613 | 63,148 | 1,601 | 9 | 172 | 5,188 |
|  | *O. orca* | 20,246 | 21,210 | 62,793 | 1,603 | 9 | 174 | 5,434 |
|  | *P. catodon* | 20,094 | 21,209 | 65,816 | 1,560 | 9 | 172 | 5,788 |
|  | *T. truncatus* | 20,309 | 21,317 | 62,146 | 1,602 | 9 | 174 | 5,419 |
| RNA-seq |  | 16,378 | 68,822 | 41,065 | 6,213 | 16 | 388 | 2,270 |
| Final set |  | 22,814 | 36,167 | 64,616 | 2,035 | 10 | 211 | 5,053 |

**Table S6. The evidence supporting gene models of the East Asian finless porpoise genome.**

|  | >=30% overlap | | >=50% overlap | | >=80% overlap | |
| --- | --- | --- | --- | --- | --- | --- |
| Overlap | **No.** | **Ratio (%)** | **No.** | **Ratio (%)** | **No.** | **Ratio (%)** |
| C (single) | 673 | 2.95 | 675 | 2.96 | 681 | 2.99 |
| H (single) | 1,193 | 5.23 | 1,440 | 6.31 | 2,214 | 9.70 |
| H (more) | 2,988 | 13.10 | 3,733 | 16.36 | 5,832 | 25.56 |
| HC | 1,156 | 5.07 | 1,592 | 6.98 | 3,748 | 16.43 |
| PC | 10 | 0.04 | 8 | 0.04 | 4 | 0.02 |
| PH | 7,007 | 30.71 | 6,481 | 28.41 | 4,569 | 20.03 |
| PHC | 9,787 | 42.90 | 8,885 | 38.95 | 5,766 | 25.27 |
| Total | 22,814 | 100.00 | 22,814 | 100.00 | 22,814 | 100.00 |

Note: “C” refers to evidences from *de novo* prediction; “H” refers to evidences from homolog prediction; “P” refers to evidences from transcriptomic prediction; “HC” refers to evidences both from *de novo* prediction and homolog prediction; “PC” refers to evidences both from transcriptomic prediction and *de novo* prediction; “PH” refers to evidences both from transcriptomic prediction and homolog prediction; “PHC” refers to evidences from transcriptomic prediction, homolog prediction and *de novo* prediction ; “single” refers to one type of evidence; “more” refers to more than one evidence. “>=30% overlap” refers to 30% of the query sequences are aligned onto target sequences; “>=50% overlap” refers to 50% of the query sequences are aligned onto target sequences; “>=80% overlap” refers to 80% of the query sequences are aligned onto target sequences.

**Table S7. Statistics of East Asian finless porpoise genes with functional classification by various methods.**

|  | Total | Nr | Swissprot | KEGG | KOG | TrEMBL | Interpro | GO | Overall |
| --- | --- | --- | --- | --- | --- | --- | --- | --- | --- |
| Number | 22,814 | 22,163 | 20,985 | 19,165 | 17,183 | 22,040 | 20,831 | 15,112 | 22,200 |
| Percentage (%) |  | 97.15 | 91.98 | 84.01 | 75.32 | 96.61 | 91.31 | 66.24 | 97.31 |
